# Flexible Organic Electrochemical Transistors for Energy-Efficient Neuromorphic Computing

**DOI:** 10.3390/nano14141195

**Published:** 2024-07-12

**Authors:** Li Zhu, Junchen Lin, Yixin Zhu, Jie Wu, Xiang Wan, Huabin Sun, Zhihao Yu, Yong Xu, Cheeleong Tan

**Affiliations:** 1 College of Integrated Circuit Science and Engineering, Nanjing University of Posts and Telecommunications, Nanjing 210023, China; zhuli0319@njupt.edu.cn (L.Z.); 1022223601@njupt.edu.cn (J.L.); wanxiang@njupt.edu.cn (X.W.); hbsun@njupt.edu.cn (H.S.); zhihao@njupt.edu.cn (Z.Y.); xuyong@njupt.edu.cn (Y.X.); 2 Yongjiang Laboratory (Y-LAB), Ningbo 315202, China; yixin-zhu@ylab.ac.cn; 3 Guangdong Greater Bay Area Institute of Integrated Circuit and System, Guangzhou 510535, China

**Keywords:** flexible organic transistor, low-power, artificial synapse, short-term and long-term plasticity, neuromorphic computing

## Abstract

Brain-inspired flexible neuromorphic devices are of great significance for next-generation high-efficiency wearable sensing and computing systems. In this paper, we propose a flexible organic electrochemical transistor using poly[(bithiophene)-alternate-(2,5-di(2-octyldodecyl)- 3,6-di(thienyl)-pyrrolyl pyrrolidone)] (DPPT-TT) as the organic semiconductor and poly(methyl methacrylate) (PMMA)/LiClO_4_ solid-state electrolyte as the gate dielectric layer. Under gate voltage modulation, an electric double layer (EDL) forms between the dielectric layer and the channel, allowing the device to operate at low voltages. Furthermore, by leveraging the double layer effect and electrochemical doping within the device, we successfully mimic various synaptic behaviors, including excitatory post-synaptic currents (EPSC), paired-pulse facilitation (PPF), high-pass filtering characteristics, transitions from short-term plasticity (STP) to long-term plasticity (LTP), and demonstrate its image recognition and storage capabilities in a 3 × 3 array. Importantly, the device’s electrical performance remains stable even after bending, achieving ultra-low-power consumption of 2.08 fJ per synaptic event at −0.001 V. This research may contribute to the development of ultra-low-power neuromorphic computing, biomimetic robotics, and artificial intelligence.

## 1. Introduction

The traditional von Neumann architecture computing systems exhibit characteristics of serial task execution. The physical separation between processing units and memory units limits their data processing capabilities and leads to high energy consumption [1,2,3,4]. Brain-inspired neuromorphic computing, with its high parallelism and brain-like processing capabilities, offers the advantages of efficiency and ultra-low-power consumption. It is considered an ideal approach to overcome the limitations of traditional computing systems and realize the next generation of artificial intelligence [5,6,7,8]. The human brain consists of approximately 10^15^ synapses and 10^11^ neurons interconnected, helping humans process complex problems involving interactions with the environment, all while consuming extremely low energy [7,9]. Therefore, simulating the functionality of synapses and neurons can contribute to the development of highly energy-efficient neuromorphic computing systems, offering a pathway to rapid advancements in artificial intelligence [10,11,12,13].

Currently, numerous electronic devices have been pursued to construct artificial synapses and neuromorphic systems, such as memristors, ferroelectric transistors, and electrochemical transistors [14,15,16,17,18,19]. Among them, two-terminal organic memristors possess advantages such as simple structure, low-power consumption, and high storage density [20,21]. They achieve resistive switching through the migration and storage of charge within the active layer, making them suitable for neuromorphic computing fields such as synaptic emulation, reservoir computing, and high-density data storage [22,23]. Three-terminal organic electrochemical transistors (OECTs) possess excellent biocompatibility, and their test parameters are controllable, making them advantageous for synaptic devices capable of both learning and transmission functions [5]. In the case of OECTs, the accumulation of ions at the semiconductor and dielectric layer interface will result in the formation of an electric double layer (EDL) under gate voltage, leads to a high capacitance, enabling the device to operate at lower voltages. A single biological synaptic event consumes only 1–10 pJ of energy [17]; therefore, it is imperative to use low-voltage organic electrochemical transistors to achieve ultra-low-power consumption synaptic devices. Furthermore, the gate dielectric exhibits ion migration characteristics, similar to ion transport in biological neural systems, allowing for the simulation of various synaptic properties [24].

Biological synapses in humans or animals are typically soft and flexible. Most reported artificial synapses lack flexibility, limiting their applications in the next generation of flexible electronics [25,26,27,28]. Organic transistors have long been considered promising candidates for flexible devices. Organic polymers are composed of organic molecules containing carbon and hydrogen, lacking a rigid crystal structure between molecules, making them more susceptible to bending and deformation while retaining their material properties [25]. Therefore, based on flexible organic electrochemical transistors, there are inherent advantages for wearable synaptic devices.

In this paper, we propose a flexible synaptic transistor based on the organic semiconductor DPPT-TT. Utilizing the ion migration properties of the solid-state electrolyte PMMA/LiClO_4_, we design an electric pulse stimulation scheme to simulate various synaptic functions, including excitatory post-synaptic currents (EPSC), paired-pulse facilitation (PPF), short-term plasticity (STP), long-term plasticity (LTP), and demonstrate its image recognition and storage capabilities in a 3 × 3 array. Furthermore, based on PI as a flexible substrate, flexible devices have been constructed and the electrical and synaptic performances remain essentially unchanged before and after bending. An ultra-low-power consumption of 2.08 fJ per synaptic event at −0.001 V has been achieved. This work opens up new possibilities for developing flexible, low-voltage artificial synaptic transistors and energy-efficient neuromorphic computing.

## 2. Materials and Methods

The specific preparation process is as follows: Firstly, DPPT-TT (Derthon, Shenzhen, China) was dissolved in DCB (Sigma Aldrich, St. Louis, MO, USA) at a concentration of 5 mg/mL and dissolved for 24 h at 80 °C. PMMA (Mw = 12,000, Sigma Aldrich) was dissolved in NBA (NBA, Sigma Aldrich) at a concentration of 100 mg/mL and dissolved for 48 h at 80 °C. Then, LiClO_4_ (Aladdin, Shanghai, China) was added to the PMMA solution at a weight ratio of 1% and stirred for 24 h at 80 °C. Subsequently, on ITO glass or ITO/PI substrate that had undergone plasma cleaning in a glovebox (oxygen content 1.7 ppm, water content 0.03 ppm), PMMA/LiClO_4_ and DPPT-TT solutions were sequentially spin-coated. The PMMA/LiClO_4_ was spin-coated at 1500 rpm for 60 s and annealed at 80 °C for 4 h. The DPPT-TT solution was spin-coated at 2000 rpm for 60 s and annealed at 150 °C for 1 h. Finally, under a pressure of 5 × 10^−4^ Pa, a 50 nm-thick source-drain electrode with a channel length of 150 μm and a channel width of 1200 μm was thermally evaporated on top of the DPPT-TT layer through a shadow mask.

The devices were cross-sectionally imaged using a scanning electron microscope (SEM, FEI-NOVA NANOSEM 230, Hillsboro, OR, USA). The surface morphology of the DPPT-TT film was characterized at room temperature using atomic force microscopy (AFM, Dimension Icon, Beijing, China). The electrical properties of the devices were tested under ambient conditions using a semiconductor analyzer (Agilent, Santa Clara, CA, USA, B1500A) at room temperature.

## 3. Results and Discussion

**Figure 1:**
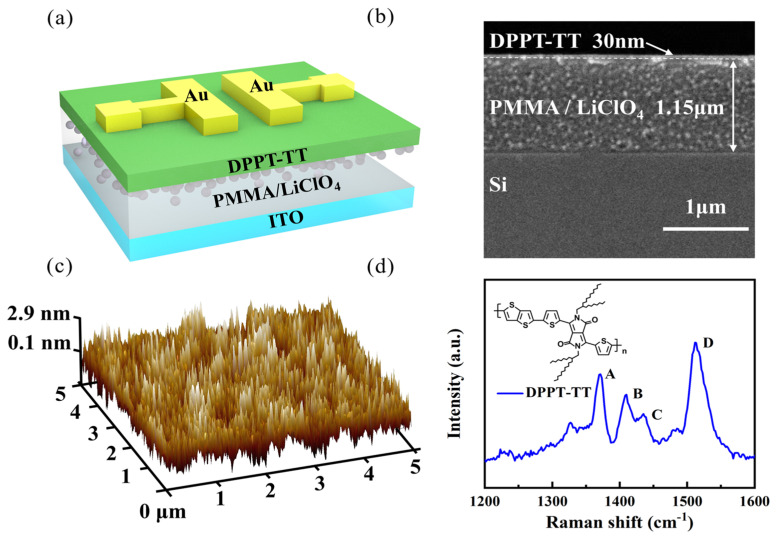
(**a**) Schematic diagram of the device structure. (**b**) Cross-sectional SEM image of the organic electrochemical transistor. (**c**) 3D AFM morphology image of the DPPT-TT film surface. (**d**) Raman spectrum of DPPT-TT, with an inset showing the molecular structure of DPPT-TT (A = 1367 cm^−1^, B = 1405 cm^−1^, C = 1432 cm^−1^, D = 1512 cm^−1^).

Figure 1a displays a schematic diagram of the bottom-gate top-contact organic electrochemical transistor (OECT) structure. Here, ITO serves as the gate electrode, PMMA/LiClO_4_ layer functions as the dielectric layer, and the gold source-drain electrodes are located on the surface of the organic semiconductor DPPT-TT film. Figure 1b presents a cross-sectional scanning electron microscopy (SEM) image of the corresponding functional film of the synaptic transistor, revealing that the thickness of the PMMA/LiClO_4_ layer is approximately 1.15 μm, while the DPPT-TT layer has a thickness of approximately 30 nm. In Figure 1c, an atomic force microscopy (AFM) 3D image of the DPPT-TT surface showcases a root mean square (rms) roughness of approximately 0.66 nm, demonstrating a smooth surface morphology conducive to constructing high-performance organic transistors. Figure 1d displays the Raman spectrum of DPPT-TT: the A peak (1367 cm^−1^) is attributed to the stretching vibration of the C-N bond within the DPP unit and the stretching vibration of the C=C double bond between units. The B peak (1405 cm^−1^) is assigned to the stretching vibration of the C=C double bond in the thiophene core. The C peak (1432 cm^−1^) corresponds to the stretching vibration of the C=C double bond in the thiophene core and the stretching vibration of the C-C bond in the thiophene ring. The D peak (1512 cm^−1^) is related to the stretching/shrinking of C=C/C-C bonds in thiophene. These results align with previous studies on the Raman spectrum of DPPT-TT [29].

**Figure 2:**
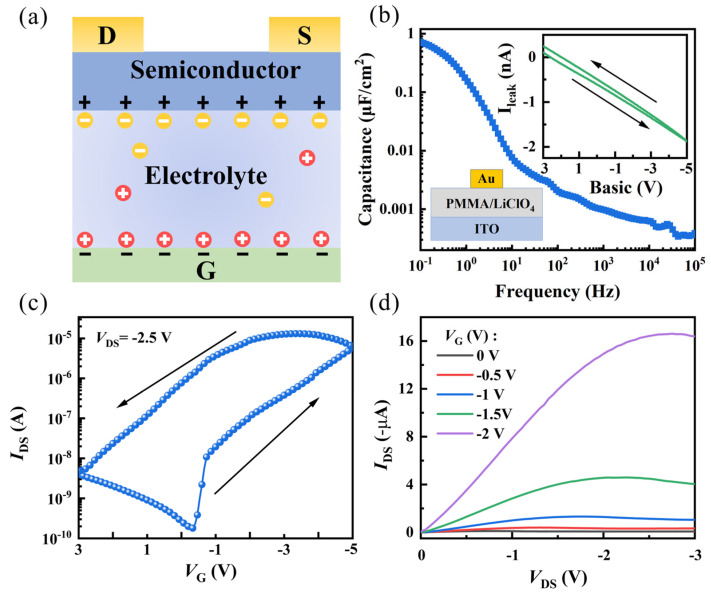
(**a**) Working mechanism of the OECT. (**b**) C–F characteristic curve of the PMMA/LiClO_4_ film, with an inset showing the I-V characteristic curve of the PMMA/LiClO_4_ film. (**c**) Transfer characteristic curve of the OECT device. (**d**) Output characteristic curve as V_GS_ varies from 0 V to −2 V.

The working mechanism of the OECT is illustrated in Figure 2a. Under the influence of the gate voltage, positive ions of the electrolyte LiClO_4_ in the dielectric layer migrate towards the gate electrode and aggregate at the interface between the dielectric layer and the gate electrode. As ions accumulate, negative ions of opposite polarity are induced to form in the gate electrode. The ITO, acting as the polarized gate electrode, does not react with the ions, thus forming an electric double layer (EDL) at the interface. Compared to regular capacitors, EDL has a smaller interlayer distance (≈1 nm), resulting in a larger capacitance [30]. Similarly, negative ions aggregate at the semiconductor layer/dielectric layer interface, allowing the device to operate at lower voltages. This aligns with the operational requirements of low-power synaptic devices.

To investigate the relationship between capacitance and frequency, as shown in Figure 2b, we prepared a capacitor using ITO as the bottom electrode and Au as the top electrode to investigate the capacitance characteristics of PMMA/LiClO_4_. The curve exhibits a notably high specific capacitance, reaching up to 0.81 μF/cm^2^ at 0.1 Hz, enabling the transistor to operate at low voltages. However, non-linear effects were observed at high frequencies. This arises from the migration of ions in response to changes in the electric field. This leads to a redistribution of charges inside the capacitor, consequently reducing the overall capacitance. Figure 2c illustrates the transfer characteristics curve of the device, displaying the characteristics of the p-type transistor. Significant counterclockwise hysteresis is observed, confirming the device’s memory effect [31]. In comparison to previous studies where pure PMMA gate dielectric layers required tens of volts for operation, this device achieves saturation current at V_GS_ = −4 V, further confirming the presence of EDL. Figure 2d presents the output characteristics curve of the device, demonstrating a good Ohmic contact. At V_DS_ = −3 V and V_GS_ = −2 V, the maximum saturation current is −16.8 μA.

**Figure 3:**
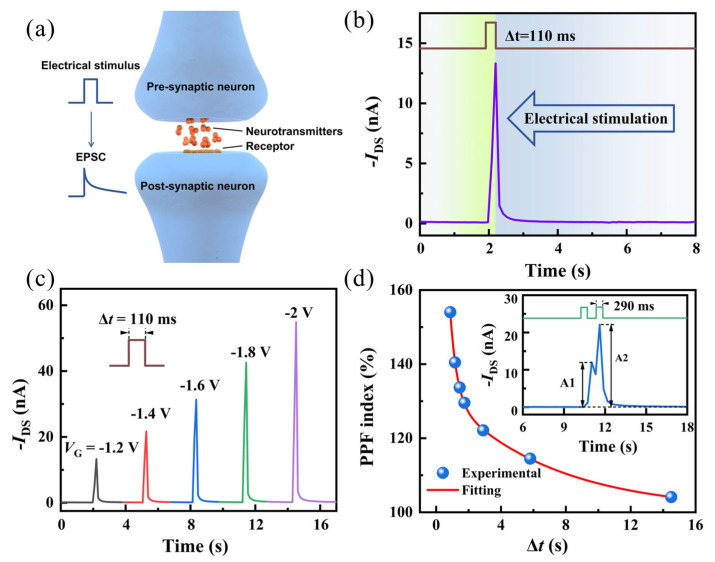
(**a**) Schematic of the artificial synapse. (**b**) Current response of the organic OECT to pulse voltage stimulation. (**c**) EPSC characteristics dependent on peak voltage at V_DS_ = −0.5 V with single-pulse triggering (V_GS_ = −2 V, 110 ms). (**d**) Function graph of PPF index (A_2_/A_1_ × 100%) as a function of time interval Δt between two electrical pulses, with an inset showing the PPF behavior triggered by two consecutive electrical pulses (−2 V, 290 ms).

In the human brain, neural networks are connected through biological synapses, where neurotransmitters transmit between pre-synaptic and post-synaptic membranes to process information, forming the physiological basis of memory with adjustable synaptic plasticity, as depicted in Figure 3a Electrochemical transistors, as shown in Figure 3b, simulate a typical EPSC response, closely resembling synaptic operations in the brain [32,33]. Human learning and memory abilities rely on the dynamic adjustment of synaptic weights in neural networks, known as synaptic plasticity, which serves as the foundation for various cognitive processes in the human brain. In general, synaptic plasticity can be classified into short-term plasticity (STP) and long-term plasticity (LTP), corresponding to learning and memory behaviors in the human brain. Here, we treat the gate electrode as the pre-synaptic neuron and the drain-source current (I_DS_) as the post-synaptic current. The typical STP characteristics of the device, EPSC, are shown first. Figure 3c displays the EPSC image at a working voltage of −0.5 V (V_DS_ = −0.5 V) and illustrates peak voltage-dependent plasticity (SVDP) characteristic. As the voltage of the pre-synaptic pulse (V_GS_ = −1.2 V to −2 V, step = −0.2 V, 110 ms) increases, the peak EPSC gradually rises from −13.32 nA to −54.92 nA. The reason for this trend is that as the presynaptic voltage increases, more negative ions gather at the dielectric layer/semiconductor interface, inducing more carriers in the semiconductor to participate in excitatory post-synaptic current.

Figure 3d demonstrates another typical STP behavior known as paired-pulse facilitation (PPF), which is believed to be linked to short-term adaptation to sensory inputs and a transient form of memory [34,35]. It can be simulated by applying two consecutive pre-synaptic pulses to the gate electrode. As shown in the inset of Figure 3d, at a working voltage of −0.5 V, with a time interval of 0.6 s between the two pulses (−2 V, 290 ms), the second pulse induces a significantly larger post-synaptic current (I_DS_) in the transistor compared to the first pulse. This is due to the second pulse inducing additional ion accumulation, indicating enhanced synaptic plasticity. This behavior can be quantified using the PPF index, defined as the ratio of the amplitudes of EPSC induced by the second and first pre-synaptic pulses (A_2_/A_1_ × 100%). As the time interval (Δt) between the two stimuli increases, the PPF index exhibits exponential decay, which can be fitted with a double exponential function [36]:

PPF ratio = 1 + B_1_exp(−Δt/τ_1_) + B_2_exp(−Δt/τ_2_) (1)

where B_1_ and B_2_ are the initial facilitation magnitudes of the rapid and slow phases, respectively. τ_1_ and τ_2_ are the characteristic relaxation times of the rapid and slow phases, respectively. And ∆t is the interval time between a pair of triggered spikes.

**Figure 4:**
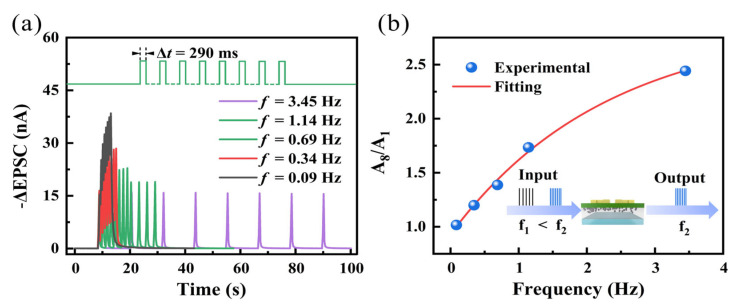
(**a**) EPSC triggered by eight consecutive pulse sequences at different frequencies (−2 V, 290 ms). (**b**) Relationship between EPSC gain triggered by consecutive electrical pulse stimulation and pulse frequency, with an inset showing the simulated high-pass filtering behavior of the device.

In biological systems, high-pass filtering is an observed phenomenon. Some synapses with lower probability of neurotransmitter release are not responsive to low-frequency pulses [37,38]. As shown in Figure 4a, under the same working voltage of −0.5 V for V_DS_, different frequency sequences of pre-synaptic pulses (8 times, −2 V, 290 ms) were applied to the transistor ranging from 0.09 Hz to 3.45 Hz. The EPSC shows significant frequency-dependent characteristics, where the peak value of EPSC (A8) is higher at higher frequencies. To evaluate the extent of EPSC enhancement induced by different frequency pulse training stimuli, we introduce the concept of EPSC gain. This is described by the ratio of EPSC amplitudes illustrated in Figure 4b. It is evident that as the pulse frequency increases, the EPSC gain becomes more pronounced. The exponential function fits the relationship between EPSC gain and pulse frequency well [39]:

EPSC amplitudes = y_0_ + C exp(−f/τ) (2)

where y_0_ is a constant C is the initial facilitation magnitudes. τ is the characteristic relaxation times. And f is the frequency corresponding to the pulse sequence. The results suggest the potential of synaptic transistors to serve as high-pass filtering elements for image preprocessing [31].

**Figure 5:**
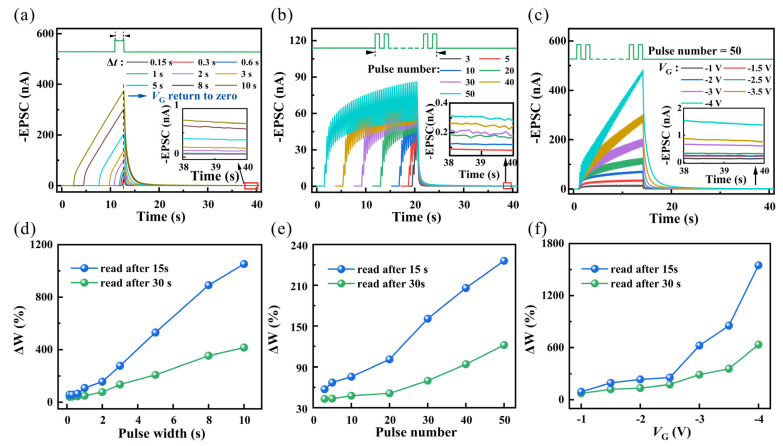
OECT device’s EPSC responses triggered by (**a**) different pulse widths, (**b**) different numbers, and (**c**) different gate voltages. Inset shows the EPSC holding value after 30 s of decay. Statistics of weight change after the stimulation ends for (**d**) different pulse widths, (**e**) different numbers, and (**f**) different gate voltages after 15 s and 30 s of decay.

In the process of brain activity, synaptic plasticity can transfer from STP to LTP under continuous stimulation. For specific neural activities, repeated stimulation can lead to a more significant enhancement of post-synaptic pulses, thereby strengthening synaptic connections [40,41]. The electrochemical transistor was subjected to different modes of stimulation at a working voltage of V_DS_ = −1 V, as illustrated in Figure 5. Figure 5a compares single pulses with different pulse widths (V_GS_ = −2 V). Clearly, shorter-duration electrical pulse stimulation leads to smaller EPSC values, and the decay rate is faster after removing the electrical pulse. After 30 s, the current decays to −80 pA, essentially returning to baseline, consistent with STP characteristics. Prolonging the duration of the electrical pulse stimulation significantly increases the EPSC values (from −24.5 nA to −374.4 nA). After the electrical pulse is removed, the EPSC of the device stimulated by a longer pulse width takes a longer time to decay back to its initial state, indicating that a longer pulse width can slow down the decay of EPSC, exhibiting a certain memory function, demonstrating the gradual transition of synaptic plasticity from STP to LTP. The same phenomenon was also observed in different numbers of consecutive pulses (−2 V, 190 ms) and a sequence of 50 consecutive pulses at different gate voltages (130 ms) (Figure 5b,c). When the number of pulses increased from 3 to 50, the corresponding maximum EPSC value of the transistor increased from −48.3 nA to −86.7 nA. When the gate voltage increased from −0.5 V to −4 V, the corresponding EPSC value increased from −15.7 nA to −483 nA. Then, the changes in synaptic weight after the end of the stimulation at 15 s and 30 s were calculated. Synaptic weight was defined as the difference between the EPSC at a certain time point and the baseline current, divided by the product of the baseline current [31]:

ΔW = (S2 − S1)/S1(3)

where S1 is the baseline current. S2 is the current maintained after forgetting. The changes in ΔW corresponding to the three stimulation modes are shown in Figure 5d–f. It is evident that relatively stronger stimulation in different stimulation modes leads to larger changes in weight values, and the decay rate of ΔW is also slower, further confirming the transition from STP to LTP.

**Figure 6:**
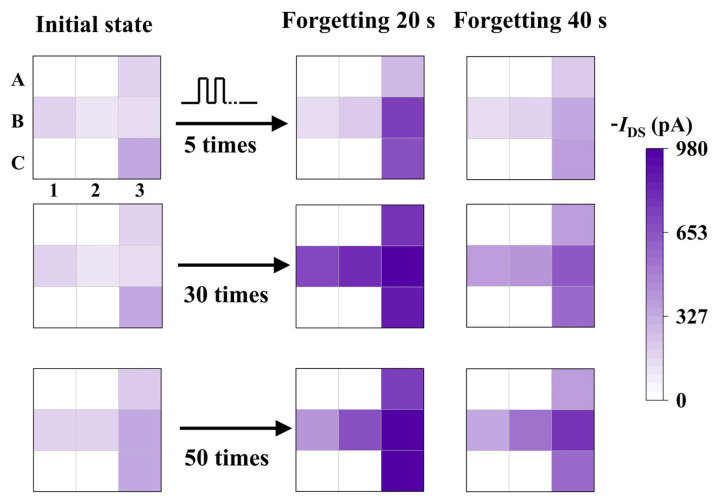
Image memory in the OECT 3 × 3 imaging array. The change in current values after stimulation with different numbers of pulses (−2 V, pulse width and pulse interval both 160 ms) under V_DS_ = −1 V.

LTP behavior holds great potential for various tasks and applications in simulating neural computing, making it crucial for neuromorphic computing [38,42]. As shown in Figure 6, a 3 × 3 array demonstrates its capabilities in image recognition and storage. Under the same pulse conditions (−2 V, 160 ms), the designed array underwent multiple pulse stimulations, specifically 5 times, 30 times, and 50 times. After 20 and 40 s following the stimulation, the current change values for each pixel in the computational imaging array were recorded. To visually assess memory retention, the color depth represents the magnitude of current change for all pixels. In each data set, it can be observed that the decay rates of different devices are nearly identical. Examining the device current at 20 s post-stimulation, it is evident that it increases with the number of pulses. The initial state current of the five devices is basically around 208.2 pA. Here, the I_DS_ under different pulse numbers for 20 s post-stimulation are presented: for 5 pulses, it is −434.8 pA; for 30 pulses, it is −646.8 pA; and for 50 pulses, it is −770.4 pA. This shows a clear enhancing trend. And the I_DS_ under different pulse numbers for 40 s post-stimulation are presented: for 5 pulses, it is −228.4 pA; for 30 pulses, it is −479.4 pA; and for 50 pulses, it is −532.2 pA. Therefore, altering the number of pulses can readily transition the array from STP to LTP or maintain its longer-lasting LTP characteristics.

**Figure 7:**
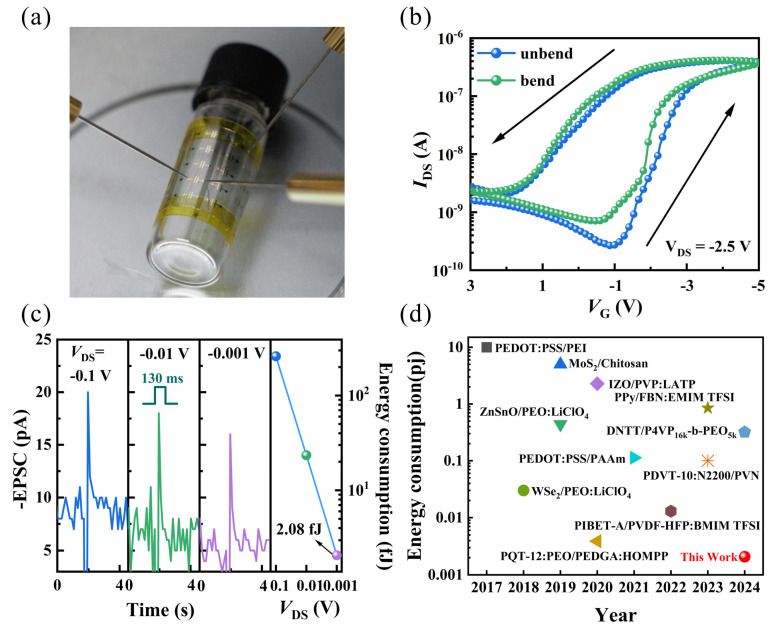
(**a**) Optical image of the OECT device in a bent state. (**b**) Transfer characteristics curves of the flexible device in bent (bending radius = 5 mm) and unbent states. (**c**) EPSC response triggered at different V_DS_ and corresponding power consumption. (**d**) The power consumption of our work is compared with that of some recently published papers [7,43,44,45,46,47,48,49,50,51,52].

To explore the application of organic electrochemical transistors (OECTs) in flexible wearable devices, transistors with the same structure were re-fabricated on ITO/PI flexible substrates. The performance of the devices before and after bending with a radius (R) is 5 mm shown in Figure 7a. The transfer characteristics curve (V_DS_ = −2.5 V) is displayed in Figure 7b, showing that the maximum current remains largely consistent before and after bending (with a difference of no more than 5%). However, after bending, the off-state current slightly increases, rising from −260 pA to −700 pA. Overall, the electrical characteristics are well maintained. More importantly, as shown in Figure 7c, the flexible device is capable of mimicking EPSC behavior at low operating voltages ranging from −0.1 V to −0.001 V. The power consumption formula for a single pulse is [5]:

P = I_DS_ × V_DS_ × Δt (4)

At a V_DS_ of −0.001 V, it achieves an ultra-low-power consumption of 2.08 fJ, demonstrating the feasibility of low-voltage operation and low-power consumption for flexible devices. Figure 7d illustrates the energy consumption of a single synaptic event in recently reported organic synaptic transistors [7,43,44,45,46,47,48,49,50,51,52], indicating significant potential for the flexible devices presented in this study to mimic the actual power consumption of a synapse in the human brain.

## 4. Conclusions

In summary, we fabricated a flexible organic electrochemical transistor with DPPT-TT as the organic semiconductor layer and PMMA/LiClO_4_ as the solid electrolyte gate dielectric layer. The presence of LiClO_4_ in the gate dielectric polymer led to synaptic behavior in the device upon applying gate bias. This successfully emulated EPSC, STP, LTP, transitions between STP and LTP, and high-pass filtering characteristics. Additionally, the existence of EDL enabled the device to operate at low voltages. The prepared flexible OECT achieved an ultra-low-power consumption of approximately 2.08 fJ per synaptic event at a working voltage of −0.001 V. As a result, it can be concluded that this study demonstrates the tremendous potential of flexible organic electrochemical transistors in neuromorphic computing systems, offering new insights into ultra-low-power wearable neuromorphic devices, biomimetic robotics, and artificial intelligence.

## Data Availability

The data presented in this study is contained within this article.
